# A re‐evaluation of the domestication bottleneck from archaeogenomic evidence

**DOI:** 10.1111/eva.12680

**Published:** 2018-09-08

**Authors:** Robin G. Allaby, Roselyn L. Ware, Logan Kistler

**Affiliations:** ^1^ School of Life Sciences University of Warwick Coventry UK; ^2^ Department of Anthropology National Museum of Natural History Smithsonian Institution Washington District of Columbia

**Keywords:** archaeogenomics, domestication, domestication bottleneck

## Abstract

Domesticated crops show a reduced level of diversity that is commonly attributed to the “domestication bottleneck”; a drastic reduction in the population size associated with subsampling the wild progenitor species and the imposition of selection pressures associated with the domestication syndrome. A prediction of the domestication bottleneck is a sharp decline in genetic diversity early in the domestication process. Surprisingly, archaeological genomes of three major annual crops do not indicate that such a drop in diversity occurred early in the domestication process. In light of this observation, we revisit the general assumption of the domestication bottleneck concept in our current understanding of the evolutionary process of domestication.

## 1 INTRODUCTION

Domestication is the process by which wild plants and animals become adapted to the human environment as a consequence of their exploitation. In plants, domestication results from the selection of a group of traits collectively termed “domestication syndrome” that make them better suited to the human environment (Harlan, De Wet, & Price, 1973). Such traits include a loss of shattering, changes in seed size, germination, and architecture (Fuller, 2007). Generally, such trait changes are disadvantageous for plants in the wild environment, but advantageous in the human environment, such that domestication is a mutualistic relationship.

**Figure 1:**
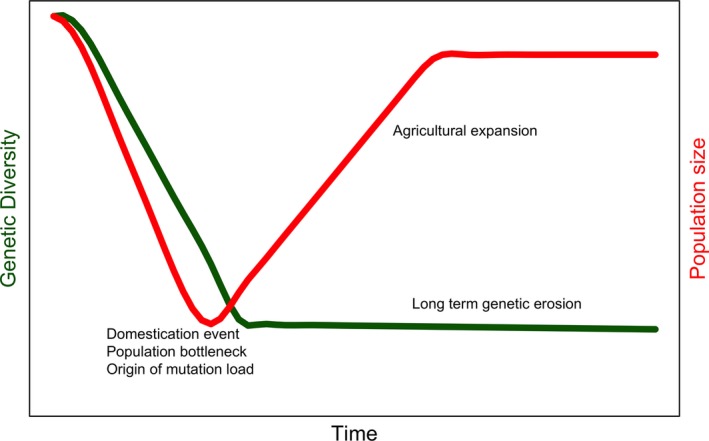
The conventional view of the domestication bottleneck

Domesticated plants generally show a significant reduction in genetic diversity relative to wild progenitor species, which is attributed to an initial dramatic reduction in population size termed the “domestication bottleneck,” followed by an expansion in population size (Meyer & Purugganan, 2013; Figure 1). This key concept has been universally applied to models of domestication (e.g., Eyre‐Walker, Gaut, Hilton, Feldman, & Gaut, 1998, Allaby, Fuller, & Brown, 2008; Zhu et al. 2007, Gaut, Díez, & Morrell, 2015) and inferred from modern genetic diversity (Meyer et al., 2016). Two key drivers underlie the domestication bottleneck concept. First, there is the notion that the domesticate population is derived from a relatively small subsample of the wild population as proto‐farmers “captured” small populations for cultivation and effectively isolated them from the wild gene pool. Second, the selection pressures involved in the transition to the domesticated form would have further reduced population sizes through the effects of the substitution load (Allaby, Kitchen, & Fuller, 2015; Haldane, 1957), originally referred to by Haldane as “the cost of selection.”

The reduction in population size associated with a bottleneck is expected to produce predictable effects. Most obviously, early population genetics theory demonstrated that the constriction in population size, if severe, would lead to a rapid loss of heterozygosity through the increased strength of drift (Nei, Maruyama, & Chakraborty, 1975). This lays the foundations for tests of population history (Tajima, 1989; Watterson, 1986), and more recently extended to the pairwise sequentially Markovian coalescent (PSMC) and the multiple sequentially Markovian coalescent (MSMC) genomewide models using the coalescent to survey the frequency of most recent common ancestor ages and reconstruct a history of population size through time (Li & Durbin, 2011; Schiffels & Durbin, 2014).

A second type of effect expected from population bottlenecks relates to the efficacy of selection. The increased strength of drift in small populations has a masking effect over selection. This can be explored by considering the strength of selection required to effect the same pace of allele frequency change as would be expected from random genetic drift representing the point at which selection is no stronger than drift (Supporting information Figure S1). We can see, for instance, that for recessive mutation in an outbreeding plant, the parameter *N*_e_ *s* (the product of effective population size and selection coefficient) is about 63.3 when drift and selection are equable. Therefore, for a selection coefficient (*s*) of 0.015, such has been found for maize (Hufford et al., 2012), effective population size (*N*_e_) would need to be considerably larger than 4,220 individuals to counter the effects of drift. The equivalent *N*_e_ *s* value for inbreeders is 3.5. Consequently, at these population sizes purifying selection begins to fail and deleterious mutations are expected to become incorporated into the population at a drift‐determined rate. Therefore, the domestication bottleneck is expected to lead to an increase in the incorporation of deleterious mutations, or the “mutation load” (Henn, Botigué, Bustamante, Clarke, & Gravel, 2015). A few studies have emerged which confirm this observation in rice (Liu, Zhou, Morrell, & Gaut, 2017), sunflower (Renaut and Rieseberg 2015), and maize (Wang et al., 2017), as well as dogs and horses in the case of animal domesticates (Marsden et al., 2016; Schubert et al., 2014). In these studies, the increased mutation load is interpreted as a cost of domestication, which is distinct to cost of selection described above consequent of the substitution load.

Intuitively, the domestication bottleneck makes sense. Notionally, we expect that domestication involved a small population that also underwent the stresses of selection; therefore, we should see reduced genetic diversity, evidence for selective sweeps, and an increased mutation load, as illustrated in Figure 1. All this evidence has been observed, which would seem to confirm the original predictions.

Over the past decade, our understanding of the process of domestication has become revised considerably (Larson et al., 2014). In the case of cereals such as rice, wheat, and barley, there has been a shift from a rapid to a protracted transition paradigm in which the emergence of domesticated forms was slow (Allaby, 2010; Allaby et al., 2008). Archaeological data show that selection for domestication syndrome traits such as loss of seed shattering and seed size was weak—comparable to that of natural selection (Purugganan & Fuller, 2009; 2011)—and that the onset of selection likely preceded domesticated forms by over ten thousand years in the case of the cereals of the Near East (Allaby, Stevens, Lucas, Maeda, & Fuller, 2017). These observations are supported by underlying theory, which suggests that under the constraints of the substitution load, there could only have been relatively few loci of selection and that weak selection would have been involved (Allaby et al., 2015). Consequently, there are relatively few opportunities for hitchhiking effects that may contribute to the mutation load. It should be noted that many of the cereals involved in domestication are inbreeders and so will tend to have relatively large levels of linkage disequilibrium increasing the Hill‐Robertson effect (Hill & Robertson, 1966)—compromising the efficiency of selection and leading to an expectation of increased mutation load. Indeed, increased mutation load has been observed in areas of the both maize (Rodgers‐Melnick et al., 2015) and rice (Liu et al., 2017) genomes in regions where recombination is lower.

The relatively slow pace of selection raises questions about the strength of selection involved and its demographic consequences on genetic diversity. Furthermore, it raises questions about the domestication bottleneck itself, and a paradox begins to emerge. If selection for the domestication syndrome was generally weak, contemporaneous, and contributory to a domestication bottleneck in which purifying selection was not efficacious leading to an increased mutation load, how could selection for the domestication syndrome have been efficacious?

A bottleneck is defined as a drastic reduction in population size, followed by a recovery. Surprisingly, the domestication bottleneck concept is one that has had little direct scrutiny, although there has been some debate as to its nature (Glemin & Bataillon, 2009). To date, the most direct tests of past bottlenecks have used the PSMC and MSMC approaches to reconstruct past population histories (Li & Durbin, 2011; Schiffels & Durbin, 2014). While such approaches have frequently been applied to human demographic history, little has yet been done to date to examine plant domestication histories. In the case of African rice, evidence for a bottleneck occurs dating to some 10,000 years prior to domestication (Meyer et al., 2016), and in the case of maize, evidence of a steady decline in diversity long predating human occupancy of the New World is observed rather than a classic bottleneck (Wang et al., 2017). Such results could reflect long‐term ecological trends, but can also reflect potentially erroneous signals if domesticate populations are derived from a subset of wild populations (Mazet, Rodríguez, Grusea, Boitard, & Chikhi, 2016).

Archaeogenomics provides a means to directly track population genomic trends through real time and so provides the most direct means to monitor the domestication bottleneck. Archaeogenomic data are available now for three major cereal crops, maize (da Fonseca et al., 2015; Ramos‐Madrigal et al., 2016; Swarts et al., 2017; Vallebueno‐Estrada et al., 2016), barley (Mascher et al., 2016), and sorghum (Smith et al., 2018), providing absolute genetic diversity measurements at points of time in the past. By taking information directly from the past, we avoid proxy estimates of past diversity based entirely on modern data. We also have the potential to negate issues arising from postdomestication crop–wild gene flow that could serve to bolster genetic diversity over time masking the effects of a domestication bottleneck, by reaching genomes before such gene flow occurred. In the case of barley, it has been shown that wild populations from across the Near Eastern region contributed to the genetic diversity of the domesticated crop (Poets, Fang, Clegg, & Morell, 2015) confounding the idea of a single point of origin for this crop (Allaby, 2015). Furthermore, archaeological evidence has been used to suggest that early domesticates were continuously replenished from wild stands, facilitating a broad gene flow into the cultivated gene pool (Willcox, 2005). Similarly, in maize, gene flow has occurred between wild and domesticated populations (Ross‐Ibarra, Tenaillon, & Baut, 2009), while sorghum has been subject to a series of transracial hybridizations giving rise to different cultivar types (Doggett, 1988).

Despite the potential complexities in the history of sorghum, Smith et al. (2018) observed a surprisingly linear decrease in individual heterozygosity over time in the case of sorghum rather than the negative exponential trend one would expect from an early initially rapid loss of diversity imposed by a domestication bottleneck. This pattern was coupled with a contrasting trend of low to high mutation load over time, which would seem to contradict the occurrence of a strong bottleneck in the earlier stages of domestication. In this study, we utilized the currently available archaeogenomic data sets of barley, maize, and sorghum to test whether the record of past diversity is compatible with the domestication bottleneck concept.

## 2 METHODS

Drift threshold values of *s* were calculated by estimating the selection coefficient required to change an allele frequency from 0.001 to 0.999 in 4*N*_e_ generations by entering dates of allele frequencies as 4*N*_e_ years apart in the selection time program (Allaby et al., 2017), for values of *N*_e_ ranging from 10 to 4,500 in increments of 10.

### 2.1 Archaeogenome data retrieval

In the case of sorghum, genomic heterozygosity data were taken directly from Smith et al. (2018). For maize, the VCF files of GBS data were used from Swarts et al. (2017). In the case of barley, VCF files for all barley accessions were obtained from Mascher et al. (2016), which included the 267 georeferenced barley landraces of Russell et al. (2016), Supporting information Table S1. While this SNP call set could be used to analyze modern barley populations, heterozygous calls were not made in the original publication for the ancient Yoram Cave barley samples. To obtain heterozygous data for the ancient barley, we therefore mapped the original ancient sequence data along with a subsample of wild and cultivated barleys (Supporting information Table S1).

The raw sequence data for ancient and modern barley were retrieved from public ENA archives in fastq format and mapped to the pseudogenome of barley utilized by Mascher et al. (2016) hosted in the e!DAL data repository. Of the Yoram Cave samples, we used sequence data sets JK2281E1U, JK2281E2U, JK3009E1U, JK3010E1U, JK3013E1U, JK3014E1U, JK3014E1U1, JK3014E1U2, JK3014E1U3, and JK3014E1U4 obtained from five ancient barley grains (JK2281, JK3009, JK3010, JK3013, and JK3014). We used sequence data sets for wild barley accessions FT11, FT037, FT045, FT067, and FT144 and for cultivated barley accessions BCC131, BCC107, BCC103, BCC105, BCC110, and BCC108. Adapters and 10 terminal bases were trimmed from the raw sequence data using cutadapt. Because the barley genome is large and highly repetitive, a mapping strategy was taken to screen out paralogous mapped reads. Initially, sequence data were split into 35nt kmers using Gerbil, and only unique 35‐mers were mapped to the barley pseudogenome using BWA (Li & Durbin, 2009) using the following parameters:

bwa aln ‐t 1 ‐i 0 ‐o 0 ‐n 0.02 ‐l 1024 ‐e 7.

SAMtools (Li et al., 2009) was used to index the barley pseudogenome and create SAM, BAM, and BCF files. BCF tools were used to convert BCF to VCFsfiles. Sites with less than 35‐fold coverage were discarded, as were 35‐mers that mapped to the genome more than once with up to a 1‐base mismatch. Sites were then further filtered to include only binary character states, 20% of taxa with data present, and a minimum mapping quality score of 50. We further used the inbreeding nature of barley as a criterion for filtering sites. A stringent cutoff of sites at which more than 25% of taxa were heterozygous was removed. This threshold was based on a binomial test of the number of heterozygous individuals expected in a population that has an inbreeding coefficient of 2/3, as determined for an 80% inbreeding system using the “estimate F” function of the selection time program (Allaby et al., 2017). This is less stringent than the expected 98% inbreeding habit of barley. We validated the appropriateness of this cutoff by comparing a range of heterozygosity thresholds and plotted resultant He and *π* values (Supporting information Figure S2). Incorporation of mismapped (paralogous) sites would be expected to inflate He estimates relative to *π*, when increasingly stringency measures cut largely legitimate sites (false negatives); then, both He and *π* estimates should reduce proportionately. We observed that a linear relationship holds between *π* and He up to a threshold of 30% heterozygous sites tolerated, above which He is disproportionately inflated relative to *π*, suggesting our 25% heterozygous cutoff is reasonable for this data set.

### 2.2 Diversity analyses

Custom scripts were used to analyze the resultant VCF files to calculate the number of heterozygous sites and empty sites to calculate the per site heterozygosity of variant sites and pairwise differences between taxa. To assess the appropriateness of using heterozygosity, we used population information from the barley and maize data sets in order to compare heterozygosity to nucleotide diversity. In the case of the maize data set, we used the populations defined in Swarts et al. (2017). In the case of the barley data set, we used the pairwise distance matrix we generated from the VCF of Mascher et al. (2016) to produce a cluster analysis using the neighbor‐joining algorithm of NEIGHBOR from the PHYLIP package (Felsenstein, 1989), from which we identified “pseudopopulations” based on clusters. We identified seven (sample sizes 42, 36, 13, 34, 16, 20 and 9) cultivated barley and two (sample sizes 23 and 18) wild pseudopopulations (Supporting information Table S1). We also considered all cultivated and all wild barleys together. For each population of maize and barley, we calculated nucleotide diversity (*π*) and individual heterozygosity (He) (Supporting information Table S1). In the case of the remapped Yoram Cave samples, we calculated *π* as the average pairwise difference between data sets obtained from different barley grains and He as the average He value per grain across data sets.

We calculated the expected reduction in genomic heterozygosity due to inbreeding from values of nucleotide diversity by determining the inbreeding coefficient F for a given level of inbreeding using the estimate F function of the selection time program (Allaby et al., 2017).

In all cases, we have used modern wild populations as an approximation of diversity at the time of domestication, which assumes that there has been little change in diversity over the Holocene.

## 3 RESULTS

For maize, we used the genotype‐by‐sequencing (GBS) data set of Swarts et al. (2017) to calculate heterozygous sites. The depth of coverage of Tehuacan maize (Ramos‐Madrigal et al., 2016) was insufficient to include in this analysis, and the data from ancient SW United States of da Fonseca et al. (2015) do not significantly overlap the GBS loci used by Swarts et al. (2017). In the case of barley, we used the data set of Mascher et al. (2016) of the 6,000‐year‐old Yoram Cave barley which included 267 modern cultivated and wild barley accessions of Russell et al. (2016). However, in the Yoram Cave data set, heterozygous sites were not called, so we remapped the ancient DNA and a sample of 11 modern wild and cultivated barleys to the same barley pseudogenome used by Mascher et al. and made heterozygous calls (see Methods).

**Figure 2:**
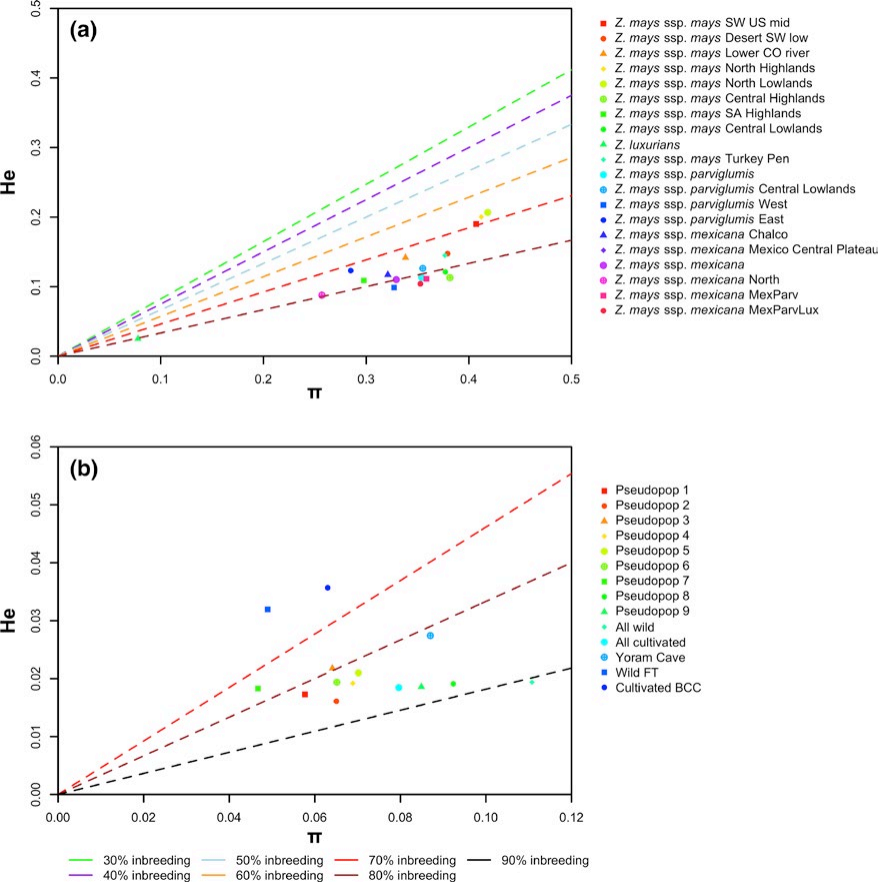
Mean population heterozygosity against population nucleotide diversity (*π*).(a) Maize populations defined by Swarts et al. (2017). (b) Barley pseudopopulations identified by cluster analysis of SNP matrix of Mascher et al. (2016). Remapped barley samples: subsample of wild accessions (FT), subsample of modern barley (BCC), and Yoram Cave ancient barley sample (Yoram Cave). Nucleotide diversity was conventionally calculated as number of pairwise differences divided by number of sites. Note in the maize data, total number of sites was unknown from GBS data so values are based on number of GBS sites reported in Swarts et al. (2017); hence, both He and *π* values are elevated

Plant accessions maintained in germplasm banks may over time suffer a loss of genomic diversity and become inbred even though the population as a whole maintains the overall nucleotide diversity. This is particularly a concern for inbreeding species such as barley. Therefore, we first assessed the use of heterozygosity as a measure of diversity by comparing the nucleotide diversity of populations with the average genomic heterozygosity of members of the same populations (Figure 2). Under ideal population conditions, nucleotide diversity is an estimator of heterozygosity. However, heterozygosity is reduced under inbreeding conditions by an extent determined by the inbreeding coefficient, *F*. We determined the expected levels of heterozygosity associated with nucleotide diversity for given levels of inbreeding (see Methods) to gauge the potential loss of diversity in germplasm samples. In the case of the maize data, suitable populations were defined by Swarts et al. (2017), Figure 2a. Despite being known as an outbreeder (Waller, 1917), maize populations showed levels of genomic heterozygosity expected of a 70%–80% inbreeder. This result could be expected if germplasm accessions had become inbred over time. However, to our surprise, the 2,000‐year‐old maize from Turkey Pen also shows this trend suggesting the possibility that this may reflect the natural ratio for maize for this data set. Many factors influence the rate of outcrossing including topography, climate, and the general ratio between self and exogenous pollen. Despite the physiological potential of a 5% inbreeding rate, the levels of inbreeding observed here have largely been observed in the field presumably due to such confounding factors (Sanvido et al., 2008). Previous studies have similarly hinted that maize may not outcross as frequently as is generally assumed (Bannert & Stamp, 2007), and evidence of extreme inbreeding has been observed directly in ancient maize genomes (Vallebueno‐Estrada et al., 2016).

For barley, we constructed pseudopopulations by clustering barley accessions on the basis of genomewide similarity (see Methods). In all, we constructed seven cultivated barley (pseudopops 1‐7) and two wild (pseudopops 8‐9) pseudopopulations (Figure 2b). Broadly, barley showed levels of heterozygosity expected of 80%–90% inbreeders, somewhat higher heterozygosity than expected for a purported 98% inbreeder. This finding is also in line with previous studies which have suggested barley may be subject to more outcrossing events than generally supposed (Morrell, Toleno, Lundy, & Clegg, 2005). In this case, the Yoram Cave samples sit in the same trend. In both the cases of barley and maize, neither modern germplasm sets show evidence of any depression in heterozygosity relative to the ancient that could be due to germplasm propagation and show that there is a consistency of signal between ancient and modern samples suggesting that heterozygosity is an appropriate measure of diversity to compare wild, modern, and ancient data sets.

**Figure 3:**
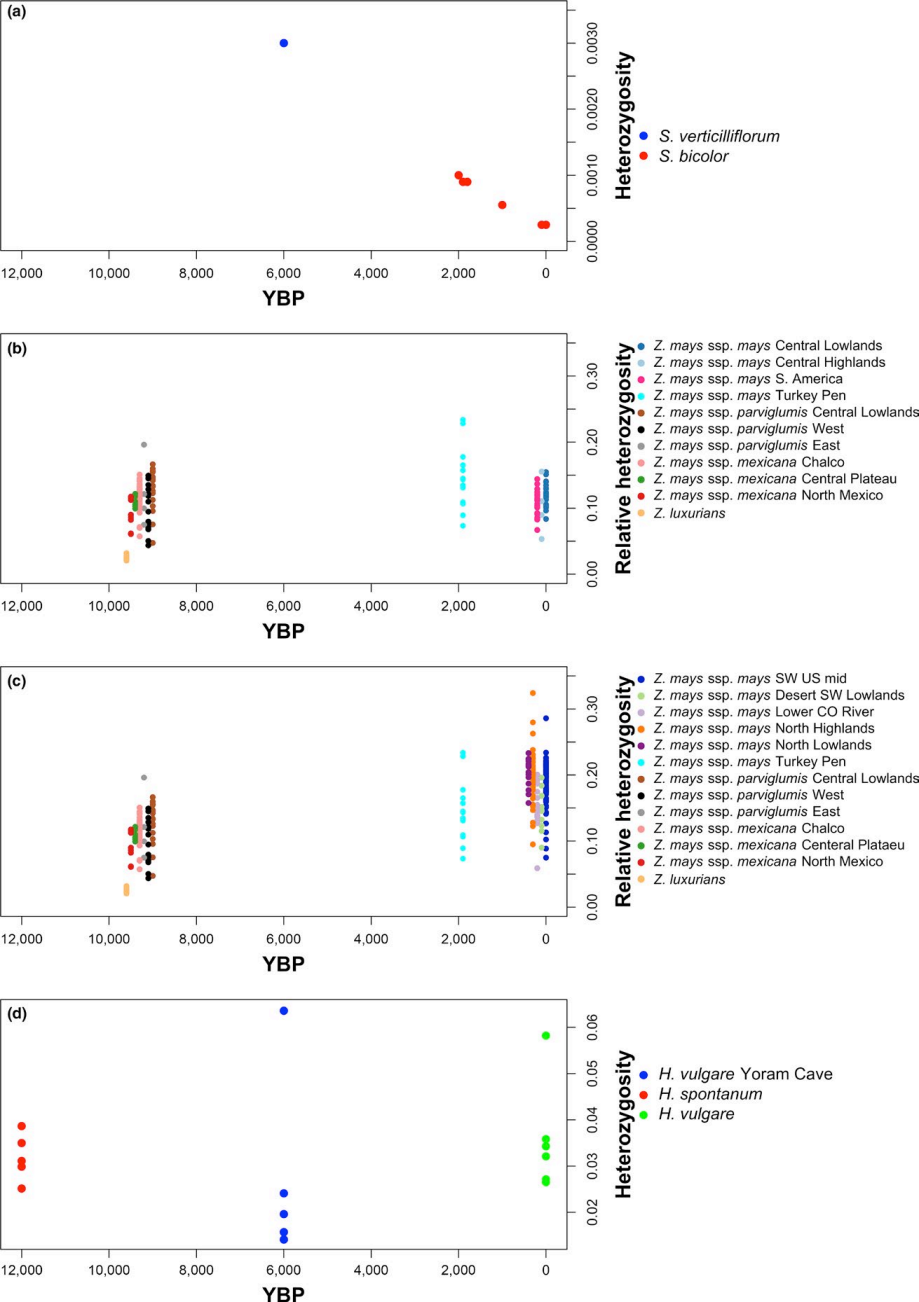
Direct estimates of genomic heterozygosity over time from modern and archaeogenomes. All heterozygosity estimates based on the number of heterozygous sites within a single genome. Modern wild progenitors taken as estimates of heterozygosity at the time of domestication. (a) *Sorghum bicolor* (data taken from Smith et al., 2018) (b) *Zea mays* of Northern Mexico and United States (data taken from Swarts et al., 2017). (c) *Zea mays* of central Mexico and South America (data taken from Swarts et al., 2017). (d) *Hordeum vulgare* (data taken from Mascher et al., 2016)

We then compared levels of heterozygosity in sorghum, maize, and barley across time (Figure 3). Maize and teosinte show a pattern that is initially counterintuitive, with some maize accessions from the US southwest and northern Mexico showing higher heterozygosity than teosinte (Figure 3b). This runs counter to the expectation of lower diversity in maize—approximately 83% relative to teosinte based on nucleotide diversity analyses (Hufford et al., 2012). This result may reflect structured populations in teosinte, which when considered together give rise to relatively high nucleotide diversity values because of the extent of interpopulation differentiation, making nucleotide diversity a poor estimator of heterozygosity in this case. Conversely, the maize of central Mexico and South America show heterozygosity similar to that found in teosinte populations (Figure 3c). It is likely the higher maize heterozygosities of northern Mexico and the United States represent accessions in which multiple teosinte populations have input genetic material augmenting the primary gene pool of domestication. Consequently, teosinte should not be considered as a single unit of origin of maize, but a series of differentiated populations, not all of which will have contributed to the maize gene pool, nor which have contributed to the diverse lineages of domestic maize uniformly. The ancient maize genomes of Turkey Pen show an elevated heterozygosity range relative to teosinte, which suggests a general reduction in heterozygosity in the central Mexican and South American maize, if continuity can be assumed, and maintenance of genetic diversity in the North Mexican and US maize.

Barley shows little change in heterozygosity over time with an increase in variation in heterozygosity apparent in the 6,000‐year‐old genomes of the Yoram Cave barley relative to the wild progenitor (Figure 3d). Similarly, the wider data set of modern wild and cultivated barleys shows no discernible difference in heterozygosity (Supporting information Figure S3). As with maize, this result runs contrary to the expectations based on species‐wide nucleotide diversity analyses that indicate a fall in diversity in landraces of about 20% (Morrell, Gonzales, Meyer, & Clegg, 2014). This discord between nucleotide diversity and heterozygosity is also likely due to the existence of structured wild populations, some of which have combined in domesticated barley, as has been previously argued (Poets et al., 2015).

In all three major crops, there is no apparent indication of the dip in genetic diversity at the individual genome level expected from a domestication bottleneck. The discord between nucleotide diversity and heterozygosity in barley and maize illustrates a requirement to rethink how genetic diversity evidence is interpreted in terms of domesticate origins. The heterozygosity evidence suggests some of the subdivided wild populations gave rise to domesticates without meaningful loss of genetic diversity. This is not a bottleneck scenario, and moreover, genetic diversity in wild populations isolated from the progenitor populations of domesticated crops should not contribute to interpretations of lost diversity during domestication.

### 3.1 Loss of genetic diversity is unlikely directly due to domestication

The lack of a dip in genetic diversity that could be associated with a distinct bottleneck episode suggests a disconnect between an intensification of selection, such as from sickle technologies (Allaby et al., 2017), and meaningful loss of genetic diversity during the rise of domesticated types. Rather, the drift‐ and selection‐associated processes responsible for the removal of diversity have acted continuously. The pattern is similar to that observed for human populations with increasing distance to Africa inversely proportional to genetic diversity, most closely modeled by a scenario of repeated bottlenecks over time driven by serial founder effects (De Giorgio, Jakobsson, & Rosenberg, 2009). Similar explanations have been put forward to explain the reduction in maize diversity over time (Wang et al., 2017). A serial founder model representative of a cropping regime where 25% grain is set aside from the harvest has also been put forward to explain loss of diversity in sorghum (Smith et al., 2018). Consequently, a simple observation of reduced genetic diversity in a domesticated crop should not be interpreted as evidence of a domestication bottleneck. The diminishing diversity observed over time reflecting a contracting effective population size may be better described as a postdomestication erosion.

A corollary of this scenario regards the mutation load, which hitherto has been regarded as a “cost of domestication.” Under a continuous erosion of genetic diversity through repeated bottlenecks, we should expect a pattern in which mutation load increases over time. This notion is supported by recent findings in which the mutation load in horses was found to increase some time after domestication (Librado et al., 2017). In the case of sorghum, mutation load was found to increase over time and significantly correlate with signals of selection (Smith et al., 2018). This could have important implications for crop management today as it would indicate that the deleterious effects of mutation load may not be the consequence of a process in the distant past, but an ongoing process that impacts crops today. It may be more accurate to describe the mutation load as a cost of agriculture or agricultural spread, rather than a cost of domestication itself.

The process of domestication cannot be pinned down to a specific point in time as different traits of the domestication syndrome were selected for at different times through changing behaviors (Fuller, Allaby, & Stevens, 2010), and even within single traits, selection pressures were dynamic over time (Allaby et al., 2017). Furthermore, a common theme from investigations in the cereals is the weakness of the selection pressures involved (Hufford et al., 2012; Purugganan & Fuller, 2011). Such weak selection requires long periods of time and a sufficiently large population to both counter the effects of drift and generate the variation required that is not represented in standing variation. The observation here of a lack of a domestication bottleneck in three major cereals suggests a resolution to the paradox that such weak selection would be unlikely to endure or contribute to a domestication bottleneck. Instead, domestication in these cases fits with a long‐term evolutionary trajectory in which genetic diversity is whittled away and mutation load accumulates through a series of minor founder events either through neutral demographic change or dynamic episodes of selection.

### 3.2 Is the domestication bottleneck a myth for annual species?

It has been observed in recent years that the perennial crops apple and grape do not show evidence of a domestication bottleneck (Gross, Henk, Richards, Fazio, & Volk, 2014; Zhou, Massonnet, Sanjak, Cantu, & Gaut, 2017), and that has been attributed to the contrasting life history of perennials exploited for domestication relative to annuals (Gaut et al., 2015). The emerging evidence from archaeogenomics suggests that annuals too may not show a strict domestication bottleneck. In the cases of barley and North American maize, as with apple and grapevine, there is hardly a discernible reduction in genetic diversity at all. While the data are currently most comprehensive for sorghum, a wider archaeogenomic survey is required across the annual domesticated plant species to establish whether the emergent picture is exceptional or a general rule.

## CONFLICT OF INTEREST

None declared.
