# Supplementary figures and images for: A re‐evaluation of the domestication bottleneck from archaeogenomic evidence
Source: Evol Appl. 2018 Sep 8;12(1):29–37. doi: 10.1111/eva.12680 (PMC6304682; doi:10.1111/eva.12680)

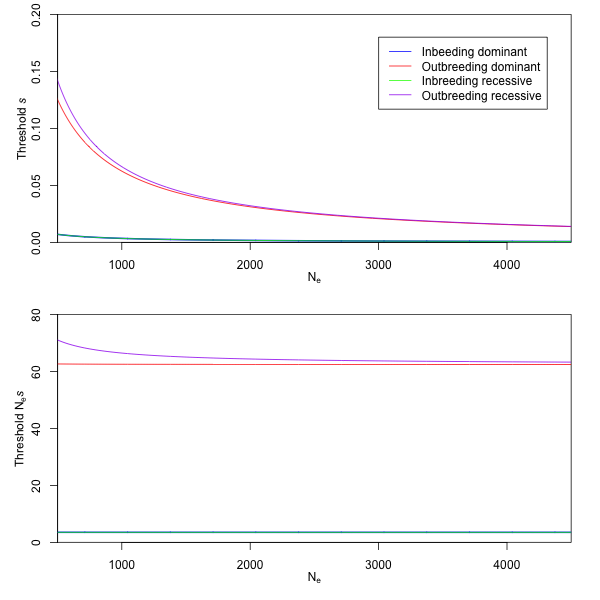

Supplement: Supplementary file 1 [file EVA-12-29-s001.png]

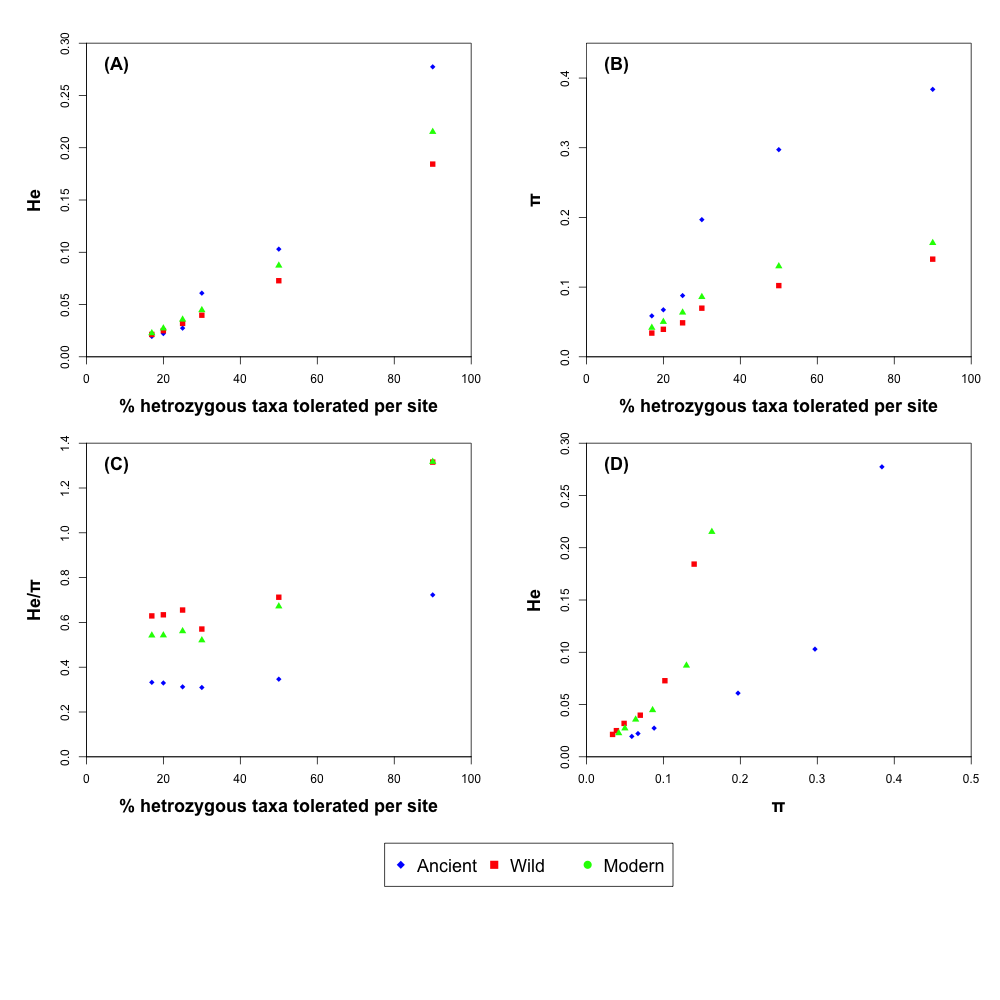

Supplement: Supplementary file 2 [file EVA-12-29-s002.png]

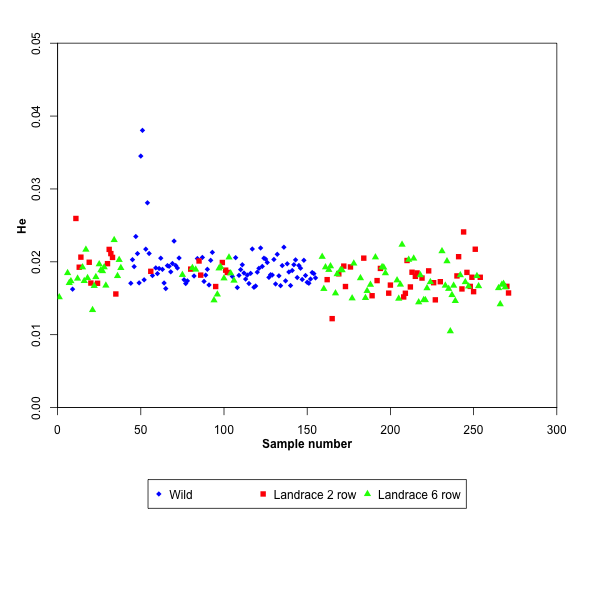

Supplement: Supplementary file 3 [file EVA-12-29-s003.png]
